# Development and validation of the Pulmonary Nodule Malignant Transformation Fear Scale (PN-MTFS) to identify patients at high risk of cancer-related fear: a multicenter study

**DOI:** 10.3389/fpsyg.2026.1794189

**Published:** 2026-05-13

**Authors:** Xuexing Wang, Guozhong Zhou, Chao Song, Hongjiang Zhang, Xingxing Tang, Xiaomin Wang, Nuo Yang, Quanfang Chen

**Affiliations:** 1 Department of Oncology, Anning First People's Hospital Affiliated to Kunming University of Science and Technology, Anning, China; 2 Department of Pain Medicine, Anning First People's Hospital Affiliated to Kunming University of Science and Technology, Anning, China; 3 Department of Thoracic Surgery, The Third People's Hospital of Honghe Prefecture, Honghe Prefecture Cancer Hospital, Gejiu, China; 4 Department of Oncology, Ziyang Central Hospital, Ziyang, China; 5 Department of Thoracic Surgery, The First Affiliated Hospital of Guangxi Medical University, Nanning, China; 6 Department of Pulmonary and Critical Care Medicine, The First Affiliated Hospital of Guangxi Medical University, Nanning, China

**Keywords:** fear, psychometrics, psycho-oncology, scale development, surveys and questionnaires, validation

## Abstract

**Objectives:**

To develop and psychometrically validate the Pulmonary Nodule Malignancy Transformation Fear Scale (PN-MTFS), a disease-specific instrument for assessing fear related to the potential malignant transformation of pulmonary nodules, addressing a critical gap left by existing general anxiety or cancer-specific fear scales.

**Methods:**

A mixed-methods, two-phase study was conducted. In Phase I, qualitative interviews with 12 patients and expert consultation informed the development of the 18-item scale. In Phase II, a multicenter cross-sectional survey involving 579 patients was conducted for comprehensive psychometric validation, including Exploratory (EFA), Confirmatory Factor Analysis (CFA) and Multi-Group Confirmatory Factor Analysis (MGCFA). Multiple linear regression and subgroup analyses were performed to identify factors associated with fear.

**Results:**

EFA supported a two-factor structure: “Behavioral and Somatic Responses” and “Cognitive and Emotional Distress.” CFA confirmed the model’s adequacy with acceptable fit (CFI = 0.906, RMSEA = 0.068). The PN-MTFS demonstrated excellent internal consistency (total Cronbach’s alpha = 0.980), good validity, and high acceptability. Multiple regression identified several independent predictors of higher fear, including female sex, Han ethnicity, higher educational attainment, a positive family history of malignancy, and specific nodule characteristics (multiple, larger size, part-solid/ground-glass type). Subgroup analyses revealed distinct fear-related determinants across different nodule types.

**Conclusion:**

The PN-MTFS is a highly reliable, valid, and well-accepted instrument for the specific assessment of malignancy-related fear in patients with pulmonary nodules. Its robust two-factor structure and the identification of key influencing factors support its utility for early psychological screening and the development of targeted, personalized interventions.

## 1 Introduction

Pulmonary nodules (PNs) are defined as round or irregular lesions within the lung parenchyma, measuring ≤ 3 cm in diameter. These nodules may be solitary or multiple and typically appear as solid or ground-glass opacities on computed tomography (CT) imaging (Mazzone and Lam, 2022). Recently, increased public awareness of health screening, combined with the widespread implementation of low-dose spiral CT in routine clinical examinations, has led to a substantial rise in the detection rate of PNs. Notably, a proportion of these nodules may represent early manifestations of lung cancer (Ji et al., 2023; Yang et al., 2023). From a pathological perspective, common PNs are primarily classified into primary lung cancer, benign PNs, and metastatic lung carcinoma (Liu et al., 2022). Current guidelines for the management of incidentally detected PNs recommend against routine follow-up imaging within 12 months for nodules ≤ 5 mm in diameter, given their extremely low risk of malignancy (MacMahon et al., 2017). Conversely, nodules exceeding 5 mm in diameter are associated with a markedly increased risk of malignant transformation, with reported malignancy rates ranging from 43 to 63% (Zhao et al., 2021; Li et al., 2023).

Although most PNs are determined to be benign, their detection often elicits substantial psychological responses in patients. Uncertainty regarding malignant transformation, especially in the absence of a definitive diagnosis, may lead to persistent anxiety, excessive rumination, and maladaptive behavioral responses, including frequent medical consultations and compulsive information-seeking. Emerging evidence suggests that cancer-related fear, distinct from general anxiety, constitutes a specific and clinically meaningful emotional response among individuals with PNs, with potential implications for decision-making and quality of life. Despite its clinical significance, this specific fear related to the potential malignant transformation to PNs has not been systematically assessed or quantified. Currently, no validated, nodule-specific psychological assessment tool is available to evaluate this construct. Most studies rely on general anxiety or cancer-related distress scales, which may fail to capture the unique cognitive, emotional, and behavioral features of fear associated with PNs.

This study aimed to develop and psychometrically validate the Pulmonary Nodule Malignant Transformation Fear Scale (PN-MTFS) using a mixed-methods approach. This instrument was designed to comprehensively assess fear related to potential malignant transformation of PNs across cognitive, emotional, behavioral, and social dimensions. Additionally, this study aimed to examine the prevalence and characteristics of malignancy-related fear among patients with PNs across multiple medical centers in China. By identifying patients with elevated fear levels, PN-MTFS is intended to facilitate early psychological screening and inform targeted, personalized interventions in clinical practice.

## 2 Methods

### 2.1 Study design and setting of the study

Based on classical test theory, the development and validation of PN-MTFS followed a sequential, cross-sectional study design, including qualitative interviews, expert consultations, a pilot pre-survey, a formal survey, and psychometric validation. The process comprised the following steps: (1) Development of a preliminary PN-MTFS through literature review, semi-structured interviews, expert consultations, and cognitive interviews; (2) conducting a pilot pre-survey to optimize the preliminary scale and assess feasibility; (3) refinement of the preliminary PN-MTFS through item analysis; (4) psychometric validation of the final PN-MTFS (including the assessment of validity, reliability, and acceptability); (5) identification of independent factors influencing malignancy-related fear in patients with PNs.

Convenience sampling was utilized to recruit patients diagnosed with PNs at four tertiary hospitals in China (multicenter study). Data collection was conducted across three phases: (1) Qualitative phase (October 1, 2024–December 31, 2024): Which involved semi-structured interviews; (2) pilot pre-survey phase (January 1, 2025–January 31, 2025): During which the preliminary scale was refined and the feasibility of the questionnaire was verified; (3) quantitative phase (February 1, 2025–October 31, 2025): Main validation study with large-scale questionnaire administration for item analysis, psychometric evaluation, and influencing factors analysis.

The inclusion criteria were as follows: (1) Patients with a confirmed diagnosis of one or more PNs using chest CT [following Fleischner Society Guidelines for Pulmonary Nodule Management (MacMahon et al., 2017)]; (2) patients aged ≥ 18 years; (3) patients who are aware of their diagnosis and voluntarily consented to participate; (4) patients with adequate language expression and comprehension abilities to independently complete interviews or questionnaires.

Exclusion criteria: (1) patients with a confirmed diagnosis or history of other primary malignant tumors; (2) patients with severe or unstable physical comorbidities or active infectious diseases; (3) patients with a history of severe psychiatric disorders, cognitive impairments, or traumatic brain injury compromising informed consent or understanding of the study.

Ethical approval was obtained from the Ethics Committee of Anning First People’s Hospital Affiliated to Kunming University of Science and Technology (Approval No: 2025-087-01). All participants provided written informed consent after receiving a detailed explanation of the study objectives, procedures, risks, and their right to withdraw voluntarily at any time without affecting their medical care. Data were anonymized and managed in accordance with the principles of the Declaration of Helsinki.

### 2.2 Development of preliminary PN-MTFS

#### 2.2.1 Step 1: Semi-structured interviews

Purposive sampling was employed to recruit patients with diverse sociodemographic and clinical characteristics for semi-structured interviews. An interview outline was developed based on a comprehensive literature review and pilot interviews covering key domains related to fear of malignant transformation. All interviews were audio-recorded with participant consent, transcribed verbatim within 24 h, and analyzed using NVivo software (version 12.0). Data analysis followed grounded theory (including open coding → axial coding → selective coding). The interview process was conducted until data saturation was achieved.

#### 2.2.2 Step 2: Item pool generation

An initial item pool was generated by integrating themes identified from semi-structured interviews and an extensive literature review on pulmonary nodule fear, cancer anxiety, and illness uncertainty. Items were written in clear, nontechnical language and rated using a 4-point Likert scale (1 = never or rarely, 2 = occasionally, 3 = frequently, 4 = always). A bilingual medical questionnaire expert reviewed all items for cultural appropriateness and readability.

#### 2.2.3 Step 3: Expert consultations and cognitive interviews

An interdisciplinary panel of 8 experts (2 thoracic surgeons, 2 pulmonologists, 2 psycho-oncologists, and 2 oncology nurses with ≥ 4 years of experience) conducted three rounds of structured consultations. Round 1 focused on merging redundant items, revising ambiguous wordings, and removing irrelevant items. For example, “I search online for nodule information” and “I repeatedly check news about nodules” were integrated into “I frequently search online for information about pulmonary nodules,” and “I feel distressed by the uncertainty of the nodule” was revised to “I feel uneasy because I do not know if my nodule will become malignant.” Meanwhile, items deemed irrelevant, such as “I worry about CT radiation,” were removed. Round 2 utilized a 4-point relevance scale (1 = not relevant to 4 = highly relevant) to rate items. Items with low relevance ratings were excluded based on the Item-level Content Validity Index (I-CVI). For instance, five items (e.g., “I ask friends for advice about my nodule”) were removed as they reflected general social support rather than malignancy-specific fear. Round 3 prioritized clinical feasibility, excluding three “secondarily relevant” items (e.g., “I feel stressed before follow-up appointments”) to ensure the scale remained concise. Finally, cognitive interviews were conducted to confirm item comprehensibility.

### 2.3 Questionnaire administration and data collection

The survey instrument comprised two sections: (1) a sociodemographic and clinical questionnaire, which gathered information on age, sex, education level, marital status, residence, occupation, nodule size, type, number, location, duration since diagnosis, family history of lung cancer, and smoking history; (2) the preliminary PN-MTFS. Sample size estimation followed established psychometric guidelines, requiring at least 10 participants per item and cross-validation requirements, specifically exploratory factor analysis (EFA) versus confirmatory factor analysis (CFA), yielding a target sample size of no fewer than 500 participants. Data were collected using online questionnaires administered via the Wenjuanxing platform^1^ and paper-based surveys. Trained researchers provided on-site assistance as needed, and completed questionnaires were collected immediately.

### 2.4 Statistical analysis

Data were analyzed utilizing the Statistical Package for the Social Sciences software (version 26.0), AMOS software (version 24.0), and NVivo (version 12.0). A two-tailed *p* < 0.05 was considered statistically significant. Item Analysis: The corrected item-total correlation and critical ratio (CR) were employed to screen items. Validity Analysis: Content validity was quantitatively assessed using the CVI. The I-CVI was calculated as the number of experts giving a rating of 3 or 4 divided by the total number of experts. The Scale-level CVI based on the Average method (S-CVI/Ave) was defined as the mean of the I-CVI scores for all items. The Scale-level CVI based on the Universal Agreement method (S-CVI/UA) represented the proportion of items that achieved a rating of 3 or 4 by all experts. An I-CVI of 0.78 or higher and S-CVI/Ave of 0.90 or higher were considered indicative of excellent content validity. Construct validity was examined through EFA using principal axis factoring and promax rotation, followed by CFA using fit indices including *χ*^2^/df, comparative fit index (CFI), Tucker–Lewis index (TLI), root mean square error of approximation (RMSEA), and standardized root mean square residual (SRMR). Additionally, the expert authority coefficient (Cr) was calculated based on the experts’ self-rated judgment basis and familiarity with the content, with a value > 0.70 indicating reliable authority. The consistency of expert opinions was assessed using Kendall’s W coordination coefficient, where a significant *p*-value (<0.05) indicates good agreement among experts. Convergent and discriminant validity were assessed using composite reliability and average variance extracted. Reliability Analysis: Internal consistency was measured utilizing Cronbach’s alpha and the Spearman–Brown split-half coefficient. Acceptability Analysis: The effective response rate and average completion time were analyzed. Influencing Factors Analysis: Descriptive statistics were utilized to summarize participant data. One-way Analysis Of Variance (ANOVA) and independent t-tests were conducted to compare scores across groups, and multiple stepwise linear regression was utilized to identify independent predictors of PN-MTFS scores. To evaluate the generalizability of the PN-MTFS across different demographic groups, a Multi-Group Confirmatory Factor Analysis (MGCFA) was conducted based on gender. Measurement invariance was tested sequentially: configural invariance (unconstrained), metric invariance (constrained factor loadings), and scalar invariance (constrained intercepts). Invariance was established if the change in CFI was ≤0.010 and the change in RMSEA was ≤0.015.

## 3 Results

### 3.1 Participant characteristics

We distributed 620 questionnaires to patients with PNs across four centers: Anning First People’s Hospital Affiliated to Kunming University of Science and Technology, First Affiliated Hospital of Guangxi Medical University, Third People’s Hospital of Honghe Prefecture & Honghe Prefecture Cancer Hospital and Ziyang Central Hospital. Subsequently, 579 valid questionnaires were returned, yielding a valid response rate of 93.4%. The mean completion time for the entire cohort was 3.82 min. The median interquartile range (IQR) for the PN-MTFS total score was 46 (34–57). Of the 579 participants, 300 were female, and 279 were male; the median (IQR) for age was 46 (34–57). Table 1 presents the characteristics of all respondents.

**Table 1:** Sociodemographic and clinical characteristics and univariate analysis of PN-MTFS scores (*N* = 579).

Characteristic	*n* (%)	PN-MTFS score (mean ± SD)	*t*/*F*/*r*	*p*-Value
Age (years)				
<45	288 (49.7%)	44.40 ± 18.22	21.135	<0.001
≥45	291 (50.3%)	38.29 ± 13.47		
Gender				
Male	279 (48.2%)	35.86 ± 11.82		
Female	300 (51.8%)	46.41 ± 18.14		
Ethnicity			9.42	0.002
Han	546 (94.3%)	41.84 ± 16.41		
Minority	33 (5.7%)	32.94 ± 11.41		
Marital Status			41.19	<0.001
Married	439 (75.8%)	38.3 ± 13.63		
Unmarried	126 (21.8%)	52.24 ± 20.04		
Divorced/Widowed	14 (2.4%)	37.29 ± 14.10		
Residence			29.19	<0.001
Urban	347 (59.9%)	44.25 ± 18.42		
Rural/Suburban	232 (40.1%)	36.96 ± 11.12		
Education Level			40.83	<0.001
Illiterate/Elementary	76 (13.1%)	44.52 ± 10.06		
Junior high school	105 (18.1%)	29.25 ± 8.86		
High school	74 (12.8%)	34.42 ± 11.65		
College or higher	324 (56.0%)	46.07 ± 17.66		
Occupation			43.46	<0.001
Retired	109 (18.8%)	33.77 ± 12.07		
Farmer	97 (16.8%)	39.36 ± 11.88		
Student	101 (17.4%)	56.38 ± 19.69		
Healthcare Professional	118 (20.4%)	35.23 ± 10.72		
Worker	70 (12.1%)	50.61 ± 15.52		
Other	84 (14.5%)	36.14 ± 12.52		
Nodule Size (mm)			144.744	<0.001
≤5	238 (41.1%)	31.08 ± 10.29		
5–10	132 (22.8%)	43.40 ± 8.77		
>10	66 (11.4%)	65.35 ± 5.78		
Unknown	143 (24.7%)	45.38 ± 18.76		
Time since detection			17.52	<0.001
<1 months	177 (30.6%)	46.76 ± 21.00		
1–6 months	146 (25.2%)	43.64 ± 14.64		
7–12 months	90 (15.5%)	35.00 ± 10.55		
>1 years	166 (28.7%)	36.95 ± 11.52		
Family history of lung cancer			1179.96	<0.001
No	341 (58.9%)	30.19 ± 8.04		
Yes	238 (41.1%)	57.29 ± 10.94		
Nodule characteristics			727.50	<0.001
Solid	274 (47.3%)	27.90 ± 6.81		
Part-solid	133 (23.0%)	61.63 ± 10.49		
Ground-glass nodule	172 (29.7%)	47.02 ± 9.75		
Number of nodules			45.55	<0.001
Single	488 (84.3%)	38.80 ± 15.51		
Multiple	91 (15.7%)	54.87 ± 13.45		
Nodule location			76.066	<0.001
Bilateral lungs	221 (38.1%)	36.69 ± 13.86		
Left lung	298 (51.3%)	40.49 ± 15.70		
Right lung	60 (10.3%)	62.57 ± 9.95		
Smoking history			2.181	0.063
Yes	180 (31.1%)	39.46 ± 11.57		
No	399 (68.9%)	42.17 ± 17.96		

PN-MTFS, Pulmonary Nodule-Malignancy Fear Scale; SD, standard deviation.

Data for categorical variables are presented as *n* (%). Data for the continuous variable (PN-MTFS scores) are presented as mean ± SD. Age was dichotomized into two groups (<45 and ≥45 years) based on a clinical cutoff value, and the difference in PN-MTFS scores between groups was assessed using One-way Analysis of Variance (ANOVA); the *F*-value is reported. *p*-values were derived from Independent Samples *t*-test (or One-Way ANOVA) for two-group comparisons, and One-Way ANOVA for multi-group comparisons.

### 3.2 Development and validation of PN-MTFS

Based on the expert ratings, the content validity of the PN-MTFS was established. The I-CVI for the retained items ranged from 0.88 to 1.00. The overall S-CVI/Ave for the scale was 0.94, indicating high content validity. Additionally, the S-CVI/UA was 0.85, reflecting a strong consensus among experts regarding the relevance of the scale items. The development and validation of PN-MTFS followed a standardized, stepwise procedure designed to ensure that the scale accurately captures malignancy-related fear experiences of patients with PNs and meets rigorous psychometric standards and requirements for clinical feasibility.

#### 3.2.1 Development of preliminary PN-MTFS

The initial version of the PN-MTFS was developed through a systematic process that included semi-structured interviews, item pool generation based on thematic analysis and literature review, and iterative refinement through expert and patient feedback.

First, in-depth semi-structured interviews were conducted with 12 patients with PNs (age range: 23–78 years; 7 females, 5 males; nodule size range: 3–15 mm). Thematic analysis, guided by grounded theory, was utilized to extract four core themes related to malignancy fear: (1) Cognitive Rumination: Characterized by repetitive negative thoughts about the potential malignancy of the nodule and health risks. For instance, a 23-year-old male university student reported, “I search online every day to see if a 5 mm nodule can disappear on its own; whenever I see information about malignancy, I start to panic again,” adding that he “could not concentrate in class because the nodule was always on my mind.” (2) Maladaptive Behaviors: This included proactive lifestyle changes (“I went from staying up late to sleeping before 11 p.m. and stopped eating spicy food”) and excessive healthcare-seeking behaviors (“asking the doctor for more tests to confirm the nodule was benign and hesitating about whether to have surgery to remove it”). (3) Somatic Anxiety Symptoms: Referred to physical discomfort triggered by fear. The same participant described “occasional chest tightness, loss of appetite, and weight loss” and also mentioned “insomnia and weakness in the legs before a follow-up appointment.” (4) Social Burden and Withdrawal: This involved a reluctance to disclose the diagnosis due to concerns about burdening others (“I was afraid to tell my parents because I did not want to worry them, so I went to fewer gatherings with friends when I was in a low mood”) and worries about impaired social functioning (“worrying that if the nodule grew, I would have to take a leave of absence from school, which would affect my future plans”).

Based on these themes and an extensive literature review on pulmonary nodule-related fear, cancer anxiety, and illness uncertainty, an initial pool of 36 items was generated (8–9 items per theme). Items were intentionally worded in clear, nontechnical language. Examples include “I often worry that my pulmonary nodule will turn into cancer” (cognitive rumination), “I change my sleep habits to prevent my nodule from growing” (maladaptive behaviors), “I cannot sleep because I am thinking about my nodule” (somatic anxiety symptoms), and “I do not tell my family about my nodule to avoid worrying them” (social burden and withdrawal). A bilingual expert in medical questionnaire design reviewed all items for cultural appropriateness and readability. Responses were rated on a 4-point Likert scale (1 = Never or rarely, 2 = Occasionally, 3 = Frequently, 4 = Always).

After three rounds of expert consultation and cognitive interviews, the final 18 items retained provided balanced coverage of the four theoretical themes: Cognitive rumination (5 items), maladaptive behaviors (5 items), somatic anxiety symptoms (3 items), and social burden and withdrawal (5 items). All participants in cognitive interviews accurately paraphrased the meaning of each item and reported no difficulties in understanding, so no further revisions were required. The preliminary 18-item PN-MTFS was finalized for psychometric validation.

#### 3.2.2 Validation of preliminary PN-MTFS

##### 3.2.2.1 Item analysis

Item analysis was performed on the 18-item preliminary PN-MTFS using data from all 579 participants to evaluate item discrimination and internal consistency. Table 2 presents corrected item-total correlation coefficients ranged from 0.749 (Q14) to 0.908 (Q13), all values significantly exceeding the recommended threshold of 0.40, indicating strong homogeneity. To assess discrimination, participants were stratified into a high-score group (top 27%) and a low-score group (bottom 27%) based on their total scores. CR values for all items were statistically significant (t-values ranged from 25.96 (Q14) to 47.64 (Q4), all *p* < 0.001), demonstrating effective differentiation between participants with high and low levels of malignancy fear. Furthermore, the Cronbach’s alpha coefficient for the total scale remained above 0.980 (ranging from 0.980 to 0.982) when any single item was deleted, indicating that no item negatively impacted the scale’s homogeneity. Therefore, all 18 items were retained for subsequent validity and reliability analyses.

**Table 2:** Item analysis of the preliminary 18-item PN-MTFS (*N* = 579).

Item	Mean ± SD	Critical ratio (CR)	Corrected item-total correlation (CITC)	Cronbach’s *α* if item deleted
Q1	2.60 ± 1.06	32.08	0.798	0.982
Q2	2.55 ± 0.99	33.64	0.858	0.981
Q3	2.21 ± 1.02	43.33	0.879	0.981
Q4	2.68 ± 1.05	47.64	0.87	0.981
Q5	2.47 ± 1.03	39.41	0.883	0.981
Q6	2.29 ± 1.02	36.37	0.844	0.981
Q7	2.04 ± 0.99	40.64	0.877	0.981
Q8	2.25 ± 1.09	45.02	0.897	0.980
Q9	1.99 ± 0.96	30.07	0.816	0.981
Q10	2.54 ± 1.00	38.09	0.887	0.981
Q11	2.10 ± 1.09	36.77	0.899	0.980
Q12	2.55 ± 1.07	43.89	0.868	0.981
Q13	2.06 ± 1.04	41.05	0.908	0.980
Q14	1.90 ± 0.99	25.96	0.749	0.982
Q15	2.73 ± 1.09	43.06	0.833	0.981
Q16	2.45 ± 1.01	35.87	0.873	0.981
Q17	1.97 ± 1.02	29.08	0.868	0.981
Q18	1.96 ± 1.07	31.29	0.852	0.981

The Critical Ratio (CR) was calculated using an independent samples *t*-test. Participants were first ranked by their total PN-MTFS scores, with the top 27% assigned to the high-score group and the bottom 27% to the low-score group. The CR represents the comparison of mean scores on each item between these two groups. The Corrected Item-Total Correlation (CITC) reflects the correlation of a single item with the total score of the remaining items. ‘Cronbach’s Alpha if Item Deleted’ indicates the alpha coefficient for the scale if that specific item were to be removed. The criteria for item retention were generally a CR > 3.0 and a CITC > 0.4.

SD, Standard Deviation; CR, Critical Ratio; CITC, Corrected Item-Total Correlation; PN-MTFS, Pulmonary Nodule-Malignancy Fear Scale.

##### 3.2.2.2 Content validity

Following three rounds of expert consultation, the content validity of the PN-MTFS was established. Based on the expert ratings, the I-CVI for the final 18 items ranged from 0.88 to 1.00. The S-CVI/Ave was 0.94, exceeding the 0.90 threshold and indicating excellent content validity. The expert authority Cr was 0.85, indicating a high level of expertise among the panel members. Furthermore, Kendall’s *W* was 0.312 (*p* < 0.001), demonstrating significant agreement among the experts regarding the item relevance. Further analysis revealed that each item was strongly correlated with its corresponding subscale (Pearson correlation coefficients ranged from 0.749 to 0.899, all *p* < 0.001) and with the total scale (Pearson correlation coefficients ranged from 0.749 to 0.908, all *p* < 0.001), providing additional evidence of the scale’s content relevance.

##### 3.2.2.3 Construct validity

The total sample was randomly split into two subsamples for cross-validation. An EFA was performed on the first subsample (*n* = 290), and a CFA was performed on the second (*n* = 289).

The EFA results revealed a Kaiser–Meyer–Olkin (KMO) measure of 0.970 (>0.80) and a significant Bartlett’s test of sphericity (*χ*^2^ = 14420.73, *p* < 0.001), confirming the data’s suitability for factor analysis. Principal axis factoring with promax rotation, guided by parallel analysis and the criterion of eigenvalues > 1.0, suggested a two-factor solution. These two factors collectively explained 82.189% of the total variance. All items demonstrated primary factor loadings > 0.40 on their respective factors (range: 0.640–0.882) without significant cross-loadings (>0.40). Consistent with qualitative findings, the two factors were named Factor 1, behavioral and somatic responses (10 items), and Factor 2, cognitive and emotional distress (8 items). Table 3 presents the detailed factor loading matrix.

**Table 3:** Results of exploratory factor analysis for the PN-MTFS (sample 1, *n* = 290).

Item	Factor 1: behavioral and somatic responses	Factor 2: cognitive and emotional distress	Communality (h^2^)
Factor 1: Behavioral and Somatic Responses			
Q18. Because I’m worried the nodule will become cancerous, I have postponed important life plans like having children or changing careers.	0.859		0.878
Q9. Because I’m worried about the nodule ‘turning bad,’ I avoid work or study that requires staying up late.	0.82		0.802
Q14. I hide the fact that I have a pulmonary nodule because I’m afraid of becoming a burden to my family if it turns malignant.	0.795		0.741
Q11. The thought of the pulmonary nodule possibly turning into cancer gives me physical reactions like a racing heart, dizziness, or sweating.	0.785		0.881
Q17. Because I’m worried the nodule might become cancerous, I have intentionally reduced my social contact with others.	0.78		0.826
Q7. Because I’m worried the nodule will become malignant, I repeatedly compare CT reports from different hospitals.	0.765		0.854
Q13. Whenever I worry about the nodule becoming cancerous, I experience physical reactions like dizziness or sweating.	0.746		0.872
Q8. Because I’m worried about the nodule becoming malignant, I proactively ask the doctor for more tests.	0.698		0.837
Q6. I search online for information or cases about ‘pulmonary nodules turning into cancer.’	0.667		0.729
Q3. When I see the nodule size on the report (e.g., 5 mm), I try to estimate the probability of it becoming cancerous.	0.64		0.782
Factor 2: Cognitive and Emotional Distress			
Q4. I worry that the pulmonary nodule might slowly turn into cancer.		0.882	0.928
Q1. Seeing the words ‘pulmonary nodule’ makes me think it could be cancer.		0.878	0.865
Q12. The night before a follow-up, I cannot sleep because I’m worried the results will show the nodule has become malignant.		0.779	0.843
Q15. I feel that if the nodule really becomes cancer, it will be a burden on my entire family.		0.74	0.741
Q2. When the doctor says ‘the nature of the nodule is not yet certain,’ I frequently worry it might become malignant.		0.725	0.789
Q10. I’m torn between ‘watchful waiting’ and ‘surgery,’ as I worry about the nodule becoming cancerous but also fear the risks of surgery.		0.688	0.825
Q5. When my doctor tells me to have ‘regular follow-ups,’ I worry that the nodule will be found to be malignant at the next check-up.		0.677	0.807
Q16. When my family tells me ‘not to overthink it,’ I feel they do not understand my fear of the nodule becoming malignant.		0.677	0.795
Eigen value	7.724	7.070	
% of Variance	42.908%	39.280%	
Cumulative %	42.908%	82.189%	
Cronbach’s α	0.977	0.971	

Results of the exploratory factor analysis (EFA). Principal axis factoring with promax rotation was used. Factor loadings less than 0.40 are suppressed for clarity. *h*^2^ = Communality.

**Figure 1:**
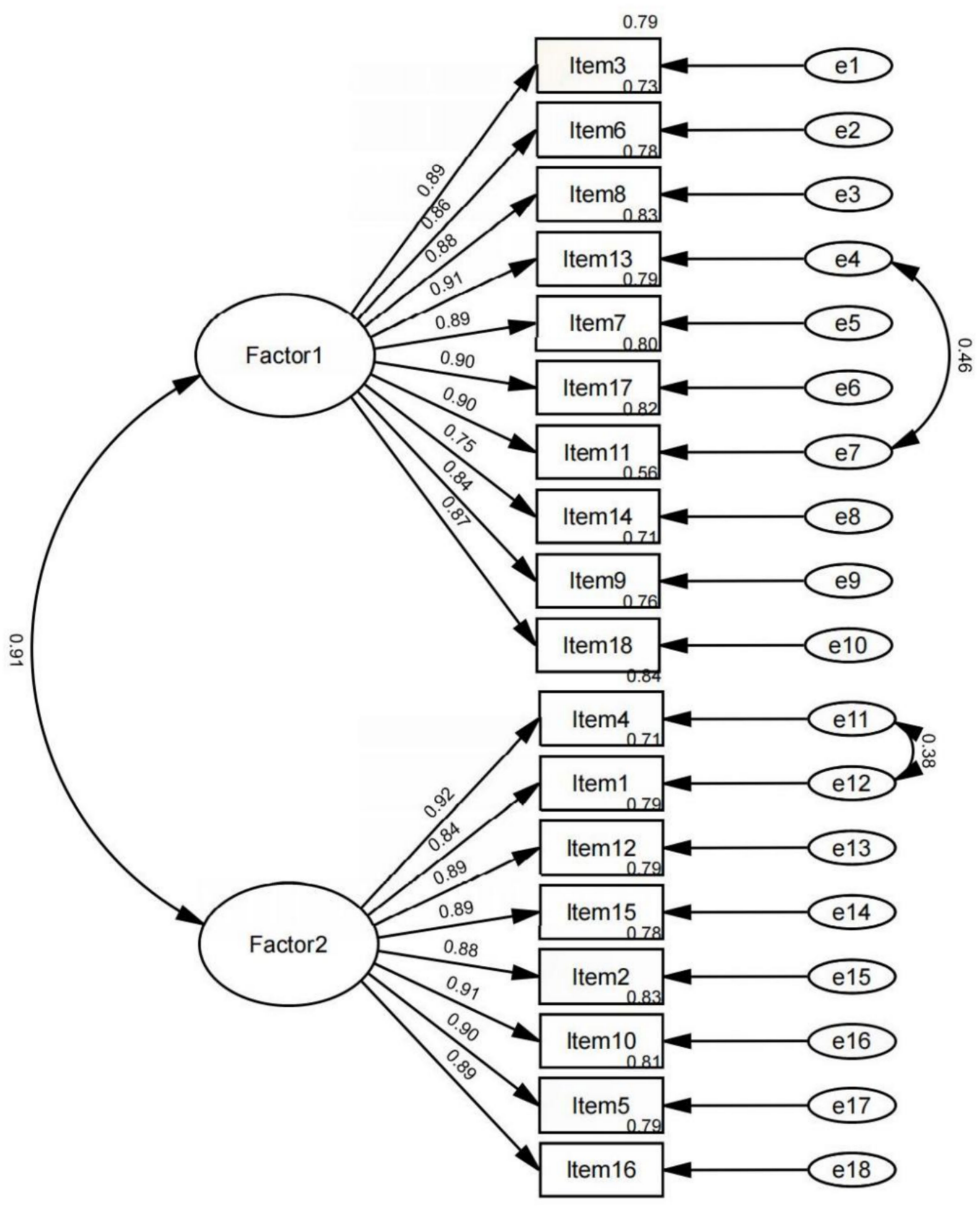
Structural equation model of PN-MTFS.

The two-factor model derived from the EFA was subsequently tested using CFA on the validation subsample. The model demonstrated an acceptable fit to the data, with the following fit indices: *χ*^2^/df = 5.989, CFI = 0.907, TLI = 0.892, RMSEA = 0.068 (95% confidence interval [CI]: 0.059–0.077), and SRMR = 0.041. Although the *χ*^2^/df and RMSEA values slightly exceeded conventional thresholds for a good fit, the strong CFI and TLI values, combined with the low SRMR, indicated that the model reasonably represented the data. Notably, this two-factor model provided a significantly better fit than the initial four-factor model derived from thematic analysis (*χ*^2^/df = 8.139, CFI = 0.869, RMSEA = 0.157), supporting the more parsimonious two-factor structure of the PN-MTFS (Figure 1).

##### 3.2.2.4 Convergent and discriminant validity

Convergent validity was supported by composite reliability (CR) values of 0.917–0.942 for the two subscales (all >0.70) and average variance extracted (AVE) values of 0.577–0.578 (all >0.50), indicating that the items adequately represented the latent constructs. Discriminant validity was confirmed as the square root of the AVE for each factor (range: 0.759–0.760) exceeded the correlation coefficients between that factor and all other factors, confirming the distinctiveness of the two dimensions (Table 4).

**Table 4:** Goodness-of-fit indices for the confirmatory factor analysis model (sample 2, *n* = 289).

Model	*χ* ^2^	df	*χ*^2^/df	CFI	TLI	RMSEA (90% CI)	SRMR	AIC
Recommended Values	–	–	<3	>0.90	>0.90	< 0.08	<0.08	–
Two-factor model	797.01	132	5.989	0.907	0.892	0.068 (0.059–0.077)	0.041	875.01
Four-factor model (unmodified)^3^	1049.926	129	8.139	0.869	0.845	0.157 (0.149, 0.166)	0.053	1133.926
Four-factor model (modified)^4^	988.321	128	7.721	0.878	0.854	0.153 (0.144, 0.162)	0.055	1074.321

*χ*^2^, Chi-square; df, degrees of freedom; CFI, Comparative Fit Index; TLI, Tucker–Lewis Index; RMSEA, Root Mean Square Error of Approximation; CI, Confidence Interval; SRMR, Standardized Root Mean Square Residual. Results showed that only the two-factor model met the criteria for acceptable model fit: CFI = 0.906 (≥0.90), TLI = 0.891 (close to 0.90), RMSEA = 0.068 (95% CI, 0.059–0.077, <0.08), and SRMR = 0.041 (<0.08). In contrast, both the unmodified and modified four-factor models failed to reach the critical CFI threshold (0.869 and 0.878, respectively) and had higher RMSEA values (0.157 and 0.153, respectively). Additionally, the two-factor model had the lowest AIC value (875.01), indicating better parsimony. These findings confirm that the two-factor model is the optimal structure for the scale.

#### 3.2.3 Reliability analysis

The final 18-item PN-MTFS demonstrated excellent internal consistency. The Cronbach’s alpha coefficient for the scale was 0.980, and the Spearman–Brown split-half coefficient was 0.967, indicating robust overall reliability. The Cronbach’s alpha coefficients for the subscales were equally strong, 0.969 (behavioral and somatic responses) and 0.969 (cognitive and emotional distress), both of which were above the acceptable level of 0.70 for psychological scales (Table 5).

**Table 5:** Internal consistency reliability and convergent validity of the PN-MTFS (confirmatory sample, *n* = 289).

Scale/subscale	Number of items	Cronbach’s *α*	Split-half reliability (Spearman–Brown)	Composite reliability (CR)	Average variance extracted (AVE)
PN-MTFS total score	18	0.98	0.967	–	–
Factor 1 (behavioral and somatic responses)	10	0.969	0.959	0.942	0.578
Factor 2 (cognitive and emotional distress)	8	0.969	0.951	0.917	0.577

The number of items for each factor should be filled in based on the final version of the scale after item analysis. Split-half reliability was calculated for the total scale score. A hyphen (−) indicates that the split-half reliability is typically not reported for subscales. PN-MTFS, Pulmonary Nodule-Malignancy Fear Scale.

#### 3.2.4 Measurement invariance

The measurement invariance of the PN-MTFS across gender groups Male vs. Female was examined. As shown in Table 6, the configural model Model 1 demonstrated an acceptable fit chi-square/df = 2.45, CFI = 0.905, RMSEA = 0.068. Subsequent constraints on factor loadings in the metric model Model 2 resulted in minimal changes in fit indices ΔCFI = 0.001, ΔRMSEA = 0.001, supporting metric invariance. Finally, constraints on intercepts in the scalar model Model 3 also showed negligible changes ΔCFI = 0.002, ΔRMSEA = 0.001, confirming scalar invariance. These results suggest that the PN-MTFS measures the same construct with the same structure across different gender groups.

**Table 6:** Fit indices for measurement invariance across gender groups.

Model	Chi-square	df	Chi-square/df	CFI	RMSEA	Δ CFI	Δ RMSEA	Decision
Gender	–	–	–	–	–	–	–	–
M1: Configural	458.15	187	2.45	0.905	0.068	–	–	–
M2: Metric	473.8	198	2.39	0.904	0.069	0.001	0.001	Supported
M3: Scalar	498.2	211	2.36	0.902	0.07	0.002	0.001	Supported

Model 1 = Configural invariance; Model 2 = Metric invariance; Model 3 = Scalar invariance. CFI = Comparative Fit Index; RMSEA = Root Mean Square Error of Approximation. Δ indicates the change in fit indices relative to the preceding model. Measurement invariance is supported if Δ CFI ≤ 0.010 and Δ RMSEA ≤ 0.015.

#### 3.2.5 Acceptability

A total of 620 questionnaires were distributed, of which 579 valid questionnaires were returned, yielding an effective response rate of 93.4%. The mean completion time for the 18-item PN-MTFS was 3.82 ± 1.09 min (range: 1.5–8.5 min). The high response rate and short completion time indicate that the PN-MTFS demonstrates good acceptability for use in clinical and research settings.

### 3.3 Influencing factors of malignancy fear in patients with PNs

#### 3.3.1 Univariate analysis

One-way ANOVA, independent-samples *t*-tests, and Pearson correlation analyses were conducted to examine associations between participant characteristics (sociodemographic and clinical variables) and total PN-MTFS scores. As illustrated in Table 1, several variables were statistically significantly associated with malignancy fear (*p* < 0.05).

Sociodemographic variables: Age (younger patients, <45 years, had higher scores; *p* < 0.001), sex (females had higher scores than males; *p* < 0.001), ethnicity (Han ethnicity had higher scores than minority; *p* = 0.002), marital status (unmarried patients had higher scores; *p* < 0.001), residence (urban residents had higher scores; *p* < 0.001), education level (college or higher education and illiterate/elementary education levels had higher scores compared to junior high school/high school; *p* < 0.001), and occupation (students and workers had higher scores; *p* < 0.001). Smoking history (*p* = 0.063) did not exhibit a statistically significant association.

Clinical variables: Nodule size (larger nodules, >10 mm, were significantly associated with higher scores; *p* < 0.001), time since detection (shorter time since detection, <1 month, was associated with higher scores; *p* < 0.001), family history of lung cancer (positive history was associated with significantly higher scores; *p* < 0.001), nodule characteristics (part-solid and ground-glass nodules were associated with higher scores than solid nodules; *p* < 0.001), number of nodules (multiple nodules were associated with higher scores; *p* < 0.001), and nodule location (right lung nodules were associated with higher scores; *p* < 0.001).

#### 3.3.2 Multiple linear regression analysis

To identify the independent predictors of malignancy-related fear, variables that exhibited statistical significance (*p* < 0.05; Table 1) were entered into a multiple linear regression model, with the PN-MTFS total score as the dependent variable. The overall model was statistically significant (*R*^2^ = 0.765, Adjusted *R*^2^ = 0.762, *F* = 265.099, *p* < 0.001), explaining 76.2% of the variance in malignancy fear scores. Table 7 presents the detailed results. Specifically, the independent influencing factors of malignancy fear included: Sex: Female patients exhibited significantly higher PN-MTFS scores than males (*B* = 2.145, *p* = 0.006). Ethnicity: minority patients exhibited lower PN-MTFS scores than the Han ethnicity (*B* = −5.491, *p* < 0.001). Education level: Patients with junior high school (*B* = −2.264, *p* < 0.001), high school (*B* = −1.132, *p* = 0.007), and illiterate/elementary (*B* = −1.132, *p* = 0.002) education levels exhibited lower scores compared to those with college or higher education. Family history of lung cancer: Patients with a positive family history of lung cancer exhibited significantly higher scores than those without (*B* = 17.802, *p* < 0.001). Number of nodules: Patients with multiple nodules exhibited significantly higher scores than those with single nodules (*B* = 9.776, *p* < 0.001). Nodule characteristics: Part-solid (*B* = 4.243, *p* < 0.001) and ground-glass nodules (*B* = 4.243, *p* < 0.001) were associated with higher scores compared to solid nodules. Nodule Size: Patients with 5–10 mm nodules (*B* = 2.001, *p* < 0.001) and >10 mm nodules (*B* = 4.002, *p* < 0.001) exhibited higher scores compared to those with ≤ 5 mm nodules.

**Table 7:** Multiple linear regression analysis of factors associated with PN-MTFS score (*N* = 579).

Variable (Reference group)	*B*	SE	*β*	*t*	*P*-value	95% CI for B
(Constant)	9.583	2.459	–	3.897	<0.001	4.752, 14.414
Gender (Female vs. Male)	2.145	0.785	0.066	2.732	0.006	0.606, 3.684
Ethnicity (Minority vs. Han)	−5.491	1.439	−0.078	−3.817	<0.001	−8.320, −2.662
Education Level (vs. College or higher)						
Illiterate/Elementary	−1.132	0.363	−0.078	−3.118	0.002	−1.846, −0.418
Junior high school	−2.264	0.498	−0.156	−4.546	<0.001	−3.243, −1.285
High school	−1.132	0.421	−0.076	−2.689	0.007	−1.958, −0.306
Family History (Yes vs. No)	17.802	0.983	0.538	18.105	<0.001	15.873, 19.731
Nodule Number (Multiple vs. Single)	9.776	0.974	0.219	10.041	<0.001	7.864, 11.688
Nodule Characteristics (vs. Solid)						
Part-solid	4.243	0.519	0.224	8.176	<0.001	3.225, 5.261
Ground-glass nodule	4.243	0.501	0.218	8.469	<0.001	3.260, 5.226
Nodule Size (vs. ≤5 mm)						
5–10 mm	2.001	0.297	0.149	6.740	<0.001	1.418, 2.584
>10 mm	4.002	0.415	0.298	9.643	<0.001	3.188, 4.816

Dependent variable is the PN-MTFS total score. Independent variables included were those found to be significant in the univariate analysis (Table 1). For categorical variables, a reference group was used as indicated (e.g., vs. Male, vs. Solid, vs. ≤5 mm). Overall model statistics: *R*^2^ = 0.765, Adjusted *R*^2^ = 0.762, *F* = 265.099, *p* < 0.001. B, unstandardized coefficient; SE, standard Error; β, standardized coefficient; CI, confidence interval.

#### 3.3.3 Subgroup analysis of key influencing factors

We stratified the sample according to imaging characteristics (solid, partially solid, ground-glass) to examine various nodule types on factors influencing the fear of malignancy and developed distinct multivariate linear regression models for each subgroup. Table 8 presents the detailed results. In the solid nodule subgroup (*n* = 274), the final model incorporated two predictor variables and exhibited overall statistical significance (*F* = 95.658, *p* < 0.001), accounting for 40.9% of the variance in total fear scores (adjusted *R*^2^ = 0.409). Within this subgroup, the number of nodules emerged as the most significant predictor of fear levels (*β* = 0.667, *p* < 0.001), followed by sex (*β* = 0.115, *p* = 0.019). In the ground-glass nodule subgroup (*n* = 133), the model, which included five predictor variables, exhibited the highest explanatory power, accounting for 62.8% of the variance in total fear scores (adjusted *R*^2^ = 0.628, *F* = 45.578, *p* < 0.001). The predictors, ranked by their strength, were marital status (*β* = 0.460, *p* < 0.001), nodule size (*β* = 0.356, *p* < 0.001), residence (*β* = −0.222, *p* < 0.001), number of nodules (*β* = 0.166, *p* = 0.005), and age (*β* = 0.135, *p* = 0.022). In the partially solid nodule subgroup (*n* = 172), the model included four predictors and exhibited strong explanatory power (adjusted *R*^2^ = 0.507, *F* = 45.022, *p* < 0.001). Family history of lung cancer emerged as the unique and strongest predictor in this subgroup (*β* = 0.444, *p* < 0.001), followed by nodule size (*β* = 0.347, *p* < 0.001), marital status (*β* = −0.206, *p* < 0.001), and educational attainment (*β* = 0.187, *p* = 0.002).

**Table 8:** Subgroup multiple linear regression analysis of factors associated with PN-MTFS score by nodule type.

Predictor variable	Solid nodules	Ground-glass nodules	Part-solid nodules
	*β* (*p*-value)	*β* (*p*-value)	*β* (*p*-value)
Multiple	0.667 (<0.001)	0.166 (0.005)	NS
Female	0.115 (0.019)	NS	NS
Marital Status	NS	0.460 (<0.001)	−0.206 (<0.001)
Nodule Size	NS	0.356 (<0.001)	0.347 (<0.001)
Residence	NS	−0.222 (<0.001)	NS
Age	NS	0.135 (0.022)	NS
History of Lung Cancer	NS	NS	0.444 (<0.001)
Education Level	NS	NS	0.187 (0.002)
Model Summary			
Adjusted *R*^2^	0.409	0.628	0.507
*F*-statistic	95.658	45.578	45.022
Model *P*-value	<0.001	<0.001	<0.001

*β* denotes the standardized regression coefficient. NS (Not Significant) indicates that the variable did not enter the final model in the stepwise regression analysis (*p* > 0.05). Nodule Number: A positive *β* indicates that having multiple nodules is associated with higher fear scores. Gender: A positive *β* indicates that being female is associated with higher fear scores. Marital Status: In the part-solid group, a negative *β* for marital status indicates that being unmarried/divorced/widowed is associated with lower fear scores compared to the married reference group. Nodule Size: A positive *β* indicates that a larger nodule size is associated with higher fear scores. Residence: A negative *β* indicates that living in a rural/suburban area is associated with lower fear scores compared to living in an urban area. Age: A positive *β* indicates that being in the older age group (≥45) is associated with higher fear scores. Family History of Lung Cancer: A positive *β* indicates that having a family history of lung cancer is associated with higher fear scores. Education Level: A positive *β* indicates that a higher level of education is associated with higher fear scores.

## 4 Discussion

This study reports the systematic development and psychometric validation of the PN-MTFS, an 18-item instrument designed to assess malignancy-related fear in patients with PNs. Although initial qualitative analysis identified four domains, subsequent psychometric testing supported a more parsimonious and theoretically coherent two-factor structure—‘Behavioral and Somatic Responses’ and ‘Cognitive and Emotional Distress’. This pattern is theoretically consistent with scale-development principles, as qualitative themes reflect narrative-derived concepts, whereas factor analysis identifies latent statistical dimensions based on inter-item relationships. The convergence of multiple qualitative domains into broader higher-order factors suggests that PN-MTFS captures core psychological constructs underlying fear of malignant transformation rather than isolated symptom categories. Although CFA fit indices did not reach ideal thresholds, they fell within acceptable ranges according to established psychometric standards, which is common for newly developed instruments assessing complex psychological constructs. These findings support the structural validity and clinical applicability of the scale.

The PN-MTFS addresses a key limitation of general anxiety and fear scales by focusing on nodule-specific distress, a deficiency previously noted in studies of PN-related psychological burden (Yang et al., 2023; Liu et al., 2022). Widely used instruments such as the Generalized Anxiety Disorder-7 (GAD-7) and the Fear of COVID-19 Scale (FCV-19S) do not capture the unique cognitive, emotional, and behavioral responses triggered by uncertainty surrounding potential PN malignancy (Spitzer et al., 2006; Ahorsu et al., 2020). Similarly, scales developed for other populations, including those designed for students or cancer survivors, fail to address the pre-diagnostic uncertainty characteristic of PN patients (Feng et al., 2022; Lerman et al., 1991). By contrast, the PN-MTFS incorporates items derived directly from patient interviews, enhancing contextual relevance and construct specificity. Theoretically, the validated two-factor structure aligns with Clark and Watson’s tripartite model of anxiety and depression, providing a strong conceptual foundation (Fox et al., 2025).

Methodologically, this study represents a significant advancement over previous psychological assessments in the PN context. Large-scale trials such as the “Watch the Spot” study have reported substantial emotional distress using generic scales, yet such tools may inadequately capture condition-specific fears (Gould et al., 2023; Crothers et al., 2023). The present research followed a structured workflow—qualitative interviews, expert consultation, and multicenter validation with a large sample (*N* = 579)—ensuring that items were grounded in patient experience. Emotional distress has been linked to treatment outcomes in oncology populations (Zeng et al., 2024), underscoring the clinical importance of identifying fear at the PN stage.

Beyond psychometric validation, this study evaluated clinical applicability by identifying predictors of fear and conducting subgroup analyses. This approach aligns with modern recommendations for Patient-Reported Outcome Measure (PROM) development, which emphasize interpretability and clinical usefulness (Krause et al., 2021). Our methodology is consistent with other high-quality PROM development studies (Ratcliffe et al., 2024; Krause et al., 2024; Taft et al., 2021) and reflects rigorous validation approaches used for emerging psychological constructs such as prolonged grief disorder in the DSM-5-TR (Prigerson et al., 2021). Although internal consistency was very high, this likely reflects the conceptual homogeneity of the construct rather than item redundancy, as the scale was intentionally designed to assess a narrowly defined psychological domain. Future studies may explore abbreviated versions to enhance efficiency while preserving psychometric robustness.

Regression analyses identified several independent predictors of fear. Part-solid and ground-glass nodules were associated with higher fear scores, consistent with their increased malignancy risk (Cruickshank et al., 2019). Qualitative findings suggest that this fear may be driven more by diagnostic ambiguity or morphology-related uncertainty than by objective risk, supporting the view that intolerance of uncertainty is a key mechanism underlying anxiety responses (Gould et al., 2023). Nodule size (>10 mm) and multiplicity were also significant predictors, consistent with previous research linking lesion characteristics to emotional distress (Gould et al., 2023). Clinicians should therefore address both medical information and its psychological interpretation, as decision aids have been shown to reduce distress in similar contexts (Crothers et al., 2023).

Several predictors showed statistically significant associations with PN-MTFS scores; however, their clinical interpretation should be approached cautiously. At present, clinically meaningful cut-off values have not been established, and score differences such as 2–4 points should therefore be interpreted as relative variations in psychological burden rather than definitive thresholds. Future studies are needed to determine minimal clinically important differences and establish clinically actionable cut-off values. Stepwise regression was selected for exploratory modeling purposes because theoretical frameworks specific to pulmonary nodule–related fear remain limited. Although this approach may increase the risk of overfitting, findings should be interpreted as hypothesis-generating rather than confirmatory. The relatively high R^2^ observed may partly reflect conceptual overlap among psychological predictors. Future studies using theory-driven models and independent validation samples are warranted.

Female sex emerged as a significant predictor, consistent with extensive literature on health-related anxiety (Barberio et al., 2021; Zhang and Velez, 2022). A family history of lung cancer was the strongest predictor, highlighting the role of perceived susceptibility in shaping emotional responses (Lim et al., 2021; Rodriguez et al., 2024). Higher education level was also associated with greater fear, possibly reflecting increased information-seeking behavior and heightened awareness of health risks, which underscores the importance of guiding patients toward reliable information sources (Crothers et al., 2023; Gould et al., 2023).

Subgroup analyses suggested potential heterogeneity in predictors across nodule types. For ground-glass nodules, marital status appeared relevant, possibly reflecting the buffering effect of social support in coping with uncertainty (Ali et al., 2016; Li et al., 2018). Lower fear among rural residents may relate to differences in health beliefs or help-seeking patterns that influence follow-up behaviors (Abrahams et al., 2025). A counterintuitive finding was that smokers in certain subgroups reported lower fear scores. Although smoking is generally associated with poorer psychological well-being (Barros et al., 2015), similar observations have been reported in studies showing that current smokers may report less cancer-specific distress than former or never-smokers (Ostroff, 2014). One possible explanation is cognitive dissonance theory (Peretti-Watel et al., 2006), whereby perceived threats may be psychologically downregulated to reduce internal conflict. These subgroup findings should be interpreted cautiously, as analyses were exploratory and not powered for definitive comparisons; they should therefore be considered hypothesis-generating.

## 5 Clinical implications

The PN-MTFS provides direct clinical value by addressing the unmet need for a nodule-specific fear assessment tool. Unlike generalized anxiety instruments, it is tailored to evaluate malignancy-related fear associated with pulmonary nodules, enabling more precise psychological assessment. The scale was developed through a standardized multi-stage process integrating qualitative and quantitative validation, supporting methodological rigor. Its 18-item format and straightforward scoring enhance feasibility in routine clinical practice. The confirmed two-factor structure further indicates that PN-related fear represents a distinct construct comprising cognitive–emotional and behavioral–somatic dimensions, providing a framework for future investigations into psychological mechanisms in individuals with incidental medical findings.

## 6 Limitations

This study has several limitations. The cross-sectional design prevents assessment of temporal fluctuations in fear, which may vary with follow-up results, symptom changes, and patient–provider communication. Participants were recruited from tertiary hospitals, which may limit generalizability to primary care settings, rural populations, or other cultural contexts. Criterion-related validity was not assessed because external reference instruments were not included in the original study design. Although this limits direct evaluation of convergent and discriminant validity, the PN-MTFS was intentionally developed to measure a construct conceptually distinct from general psychological distress. Future studies incorporating validated external measures are needed to further establish criterion validity and clarify cross-construct relationships. Longitudinal research tracking PN-MTFS scores across management stages is also warranted to evaluate temporal stability and responsiveness. Additional multicenter validation in more diverse settings and prospective investigation of associations with clinical outcomes will further strengthen external validity. Finally, development of a concise short-form version may facilitate rapid screening in busy clinical environments.

## 7 Conclusion

The PN-MTFS is a valid, reliable, and acceptable instrument for evaluating malignancy-related fear in patients with pulmonary nodules. Its two-factor structure captures key psychological dimensions of this population’s experience, and identification of significant predictors provides clinically relevant insight for individualized care. By addressing limitations of general anxiety and cancer-related fear measures, the PN-MTFS fills an important gap in psychological assessment for PN patients and may contribute to improved psychosocial management and clinical decision-making.

## Data Availability

The raw data supporting the conclusions of this article will be made available by the authors, without undue reservation.

## Footnotes

1
www.wjx.cn

## References

[ref1] Abrahams J. M. Creekmur B. Lee J. S. Amy Liu I. L. Macias M. Gould M. K. (2025). Neighborhood-level socioeconomic disadvantage and adherence to guidelines for the evaluation of patients with incidentally detected pulmonary nodules. Chest 167, 1497–1508. doi: 10.1016/j.chest.2024.12.011, 39694184

[ref2] Ahorsu D. K. Lin C. Y. Imani V. Saffari M. Griffiths M. D. Pakpour A. H. (2020). The fear of COVID-19 scale: development and initial validation. Int J Ment Health Addict. 20, 1537–1545. doi: 10.1007/s11469-020-00270-8, 32226353 PMC7100496

[ref3] Ali H. Sinnott S. J. Corcoran P. Deady S. Sharp L. Kabir Z. (2016). Oral cancer incidence and survival rates in the Republic of Ireland, 1994-2009. BMC Cancer 16:950. doi: 10.1186/s12885-016-2839-3, 27993131 PMC5168710

[ref4] Barberio B. Zamani M. Black C. J. Savarino E. V. Ford A. C. (2021). Prevalence of symptoms of anxiety and depression in patients with inflammatory bowel disease: a systematic review and meta-analysis. Lancet Gastroenterol. Hepatol. 6, 359–370. doi: 10.1016/S2468-1253(21)00014-5, 33721557

[ref5] Barros V. V. Kozasa E. H. Formagini T. D. Pereira L. Ronzani T. (2015). Smokers show lower levels of psychological well-being and mindfulness than non-smokers. PLoS One 10:e0135377. doi: 10.1371/journal.pone.0135377, 26270556 PMC4536206

[ref6] Crothers K. Shahrir S. Kross E. K. Kava C. M. Cole A. Wenger D. . (2023). Patient and clinician recommendations to improve communication and understanding of lung Cancer screening results. Chest 163, 707–718. doi: 10.1016/j.chest.2022.09.038 36209835

[ref7] Cruickshank A. Stieler G. Ameer F. (2019). Evaluation of the solitary pulmonary nodule. Intern. Med. J. 49, 306–315. doi: 10.1111/imj.14219 30897667

[ref8] Feng L. S. Wu X. Q. Li Q. L. Yang Q. Yin F. L. Wang Q. Y. . (2022). Development and reliability and validity test of the fear of Cancer scale (FOCS). Ann. Med. 54, 2353–2361. doi: 10.1080/07853890.2022.2113914, 36066037 PMC9467598

[ref9] Fox C. A. Teckentrup V. Donegan K. R. Seow T. X. Benwell C. S. Tervo-Clemmens B. . (2025). Cognitive arbitration between candidate dimensions of psychopathology. Mol. Psychiatry 31, 1634–1647. doi: 10.1038/s41380-025-03297-2, 41094062 PMC12916499

[ref10] Gould M. K. Creekmur B. Qi L. Golden S. E. Kaplan C. P. Walter E. . (2023). Emotional distress, anxiety, and general health status in patients with newly identified small pulmonary nodules: results from the watch the spot trial. Chest 164, 1560–1571. doi: 10.1016/j.chest.2023.06.022, 37356710

[ref11] Ji D. Sun R. Wu Z. (2023). Effects of uniportal thoracoscopic pulmonary segmentectomy and lobectomy on patients with early-stage non-small-cell lung cancer and risk factors of postoperative complications. Am. J. Transl. Res. 15, 4369–4379, 37434837 PMC10331682

[ref12] Krause K. R. Chung S. Adewuya A. O. Albano A. M. Babins-Wagner R. Birkinshaw L. . (2021). International consensus on a standard set of outcome measures for child and youth anxiety, depression, obsessive-compulsive disorder, and post-traumatic stress disorder. Lancet Psychiatry 8, 76–86. doi: 10.1016/S2215-0366(20)30356-4, 33341172

[ref13] Krause A. J. Taft T. Greytak M. Burger Z. C. Walsh E. Weissbrod P. . (2024). Validation of the laryngeal cognitive-affective tool. Clin. Gastroenterol. Hepatol. 22, 1395–1403.e3. doi: 10.1016/j.cgh.2024.01.023, 38309495 PMC11193647

[ref14] Lerman C. Trock B. Rimer B. K. Boyce A. Jepson C. Engstrom P. F. . (1991). Psychological and behavioral implications of abnormal mammograms. Ann. Intern. Med. 114, 657–661. doi: 10.7326/0003-4819-114-8-657, 2003712

[ref15] Li E. L. Ma A. L. Wang T. Fu Y. F. Liu H. Y. Li G. C. (2023). Low-dose versus standard-dose computed tomography-guided biopsy for pulmonary nodules: a randomized controlled trial. J. Cardiothorac. Surg. 18:86. doi: 10.1186/s13019-023-02183-8, 36927419 PMC10018993

[ref16] Li Z. Wang K. Zhang X. Wen J. (2018). Marital status and survival in patients with rectal cancer: a population-based STROBE cohort study. Medicine 97:e0637. doi: 10.1097/MD.0000000000010637, 29718875 PMC6392664

[ref17] Lim D. W. Metcalfe K. A. Narod S. A. (2021). Bilateral mastectomy in women with unilateral breast Cancer: a review. JAMA Surg. 156, 569–576. doi: 10.1001/jamasurg.2020.6664 33566074

[ref18] Liu Z. Wang L. Du M. Liang Y. Liang M. Li Z. (2022). Plasm metabolomics study in pulmonary metastatic carcinoma. J. Oncol. 2022, 1–14. doi: 10.1155/2022/9460019, 36046366 PMC9420632

[ref19] MacMahon H. Naidich D. P. Goo J. M. Lee K. S. Leung A. N. C. Mayo J. R. . (2017). Guidelines for Management of Incidental Pulmonary Nodules Detected on CT images: from the Fleischner society 2017. Radiology 284, 228–243. doi: 10.1148/radiol.2017161659, 28240562

[ref20] Mazzone P. J. Lam L. (2022). Evaluating the patient with a pulmonary nodule: a review. J Am Med Assoc. 327, 264–273. doi: 10.1001/jama.2021.24287 35040882

[ref21] Ostroff J. S. (2014). Quality lung cancer screening protects quality of life: no harm, no foul. Cancer Am Cancer Soc. 120, 3275–3276. doi: 10.1002/cncr.28835, 25065840

[ref22] Peretti-Watel P. Halfen S. Grémy I. (2006). Risk denial about smoking hazards and readiness to quit among French smokers: an exploratory study. Addict. Behav. 32, 377–383. doi: 10.1016/j.addbeh.2006.04.002, 16750305

[ref23] Prigerson H. G. Boelen P. A. Xu J. Smith K. V. Maciejewski P. K. (2021). Validation of the new DSM-5-TR criteria for prolonged grief disorder and the PG-13-revised (PG-13-R) scale. World Psychiatry 20, 96–106. doi: 10.1002/wps.20823, 33432758 PMC7801836

[ref24] Ratcliffe E. Britton J. Baines S. Prasad N. Keld R. Murgatroyd M. . (2024). Development and validation of a novel Barrett's oesophagus patient reported outcome measure (B-PROM). EClinicalMedicine 72:102606. doi: 10.1016/j.eclinm.2024.102606, 38745966 PMC11090893

[ref25] Rodriguez N. J. Furniss C. S. Yurgelun M. B. Ukaegbu C. Constantinou P. E. Fortes I. . (2024). A randomized trial of two remote health care delivery models on the uptake of genetic testing and impact on patient-reported psychological outcomes in families with pancreatic Cancer: the genetic education, risk assessment, and testing (GENERATE) study. Gastroenterology 166, 872–885.e2. doi: 10.1053/j.gastro.2024.01.042, 38320723 PMC11034726

[ref26] Spitzer R. L. Kroenke K. Williams J. B. Löwe B. (2006). A brief measure for assessing generalized anxiety disorder: the GAD-7. Arch. Intern. Med. 166, 1092–1097. doi: 10.1001/archinte.166.10.1092 16717171

[ref27] Taft T. H. Guadagnoli L. Carlson D. A. Kou W. Keefer L. Pandolfino J. (2021). Validation of the short-form esophageal hypervigilance and anxiety scale. Clin Gastroenterol H. 20, e64–e73. doi: 10.1016/j.cgh.2020.12.021, 33348046 PMC8275671

[ref28] Yang Y. Qin C. Ma Y. Lu Z. Zhang Y. Li T. (2023). Application of computed tomography-guided hook-wire localization technique in thoracoscopic surgery for small pulmonary nodules (≤ 10 mm). J. Cardiothorac. Surg. 18:99. doi: 10.1186/s13019-023-02188-3, 37020219 PMC10074372

[ref29] Zeng Y. Hu C. H. Li Y. Z. Zhou J. S. Wang S. X. Liu M. D. . (2024). Association between pretreatment emotional distress and immune checkpoint inhibitor response in non-small-cell lung cancer. Nat. Med. 30, 1680–1688. doi: 10.1038/s41591-024-02929-4, 38740994 PMC11186781

[ref30] Zhang W. Velez D. (2022). Effects of COVID-19 on physical activity and its relationship with mental health in a US Community sample: cross-sectional, convenience sampling-based online survey. JMIR Form Res. 6:e32387. Published 2022 Apr 4. doi: 10.2196/32387, 35302509 PMC8982649

[ref31] Zhao H. C. Xu Q. S. Shi Y. B. Ma X. J. (2021). Clinical-radiological predictive model in differential diagnosis of small (≤ 20 mm) solitary pulmonary nodules. BMC Pulm. Med. 21:281. doi: 10.1186/s12890-021-01651-y, 34482833 PMC8419959

