# Supplementary material for: Development and validation of the Pulmonary Nodule Malignant Transformation Fear Scale (PN-MTFS) to identify patients at high risk of cancer-related fear: a multicenter study
Source: Front Psychol. 2026 May 13;17:1794189. doi: 10.3389/fpsyg.2026.1794189 (PMC13212492; doi:10.3389/fpsyg.2026.1794189)
Supplement: Supplementary file 1 [file Supplementary_file_1.docx]

**Pulmonary Nodule-Malignancy Fear Scale (PN-MFS)**

Participant ID: _______________ Date: _______________

Instructions for Participants

Dear Participant,

Thank you for agreeing to take part in this study. This questionnaire is designed to help us understand the thoughts, feelings, and experiences of individuals who have been diagnosed with a pulmonary nodule.Your responses are very important for improving the care and support we provide to patients like you.

Please read each question carefully and provide the most accurate answers based on your personal experience. There are no right or wrong answers. All your responses will be kept strictly confidential and will be used for research purposes only. Your participation is completely voluntary, and you may withdraw at any time without any impact on your medical care.

This questionnaire consists of two parts and should take approximately 5 minutes to complete.Thank you for your valuable contribution.

**Part I: Background Information**

Please fill in the blanks or check(√) the box (□) that best describes you.

Age: ________ years

Gender: □ Male □ Female

Marital Status: □ Married □ Unmarried □ Divorced/Widowed

Residence: □ Urban □ Rural/Suburban

Highest Education Level: □ Illiterate/Elementary □ Junior high school □ High school □ College or higher

Occupation: □ Retired □ Farmer □ Student □ Healthcare Professional □ Worker □ Other

Largest Nodule Size (mm): □ ≤5 □ 5–10 □ >10 □ Unknown

Time Since Detection: □ <1 month □ 1–6 months □ 7–12 months □ >1 year

Family History of Lung Cancer: □ No □ Yes

Nodule Type: □ Solid □ Part-solid □ Ground-glass

Number of Nodules: □ Single □ Multiple

Smoking History: □ Yes □ No

**Part II: PN-MFS Questionnaire**

**Factor 1: Behavioral and Somatic Responses**

During the past month, have you had any of the following behaviors or physical feelings?

1.When you see the nodule size on your report (e.g., 5mm), have you tried to estimate the probability of it becoming cancerous?
□ Not at all      □ A little      □ Quite a bit      □ Very much

2.Have you searched online for information or cases about 'pulmonary nodules turning into cancer'?
□ Not at all      □ A little      □ Quite a bit      □ Very much

3.Because you are worried the nodule will become malignant, have you repeatedly compared CT reports from different hospitals?
□ Not at all      □ A little      □ Quite a bit      □ Very much

4.Because you are worried about the nodule becoming malignant, have you proactively asked your doctor for more tests?
□ Not at all      □ A little      □ Quite a bit      □ Very much

5.Because you are worried about the nodule 'turning bad,' have you avoided work or study that requires staying up late?
□ Not at all      □ A little      □ Quite a bit      □ Very much

6.Has the thought of the nodule possibly turning into cancer given you physical reactions (e.g., a racing heart, dizziness, or sweating)?
□ Not at all      □ A little      □ Quite a bit      □ Very much

7.When you worry about the nodule becoming cancerous, do you experience physical reactions like dizziness or sweating?
□ Not at all      □ A little      □ Quite a bit      □ Very much

8.Have you hidden the fact that you have a pulmonary nodule because you are afraid of becoming a burden to your family if it turns malignant?
□ Not at all      □ A little      □ Quite a bit      □ Very much

9.Because you are worried the nodule might become cancerous, have you intentionally reduced your social contact with others?
□ Not at all      □ A little      □ Quite a bit      □ Very much

10.Because you are worried the nodule will become cancerous, have you postponed important life plans (e.g., having children, changing careers)?
□ Not at all      □ A little      □ Quite a bit      □ Very much

**Factor 2: Cognitive and Emotional Distress**

11.During the past month, have you had any of the following thoughts or feelings?

Does seeing the words 'pulmonary nodule' make you think it could be cancer?
□ Not at all      □ A little      □ Quite a bit      □ Very much

12.When your doctor says 'the nature of the nodule is not yet certain,' have you frequently worried it might become malignant?
□ Not at all      □ A little      □ Quite a bit      □ Very much

13.Have you worried that the pulmonary nodule might slowly turn into cancer?
□ Not at all      □ A little      □ Quite a bit      □ Very much

14.When your doctor tells you to have 'regular follow-ups,' have you worried that the nodule will be found to be malignant at the next check-up?
□ Not at all      □ A little      □ Quite a bit      □ Very much

15.Have you felt torn between 'watchful waiting' and 'surgery,' as you worry about the nodule becoming cancerous but also fear the risks of surgery?
□ Not at all      □ A little      □ Quite a bit      □ Very much

16.The night before a follow-up, have you had trouble sleeping because you are worried the results will show the nodule has become malignant?
□ Not at all      □ A little      □ Quite a bit      □ Very much

17.Have you felt that if the nodule really becomes cancer, it will be a burden on your entire family?
□ Not at all      □ A little      □ Quite a bit      □ Very much

18.When your family tells you 'not to overthink it,' have you felt they do not understand your fear of the nodule becoming malignant?
□ Not at all      □ A little      □ Quite a bit      □ Very much

Thank you for your participation!
